# Food Safety Impacts from Post-Harvest Processing Procedures of Molluscan Shellfish

**DOI:** 10.3390/foods5020029

**Published:** 2016-04-18

**Authors:** George L. Baker

**Affiliations:** University of Florida, 104 Aquatic Food Products Laboratory, Gainesville, FL 32611-0370, USA; glba@ufl.edu; Tel.: +01-352-294-3902

**Keywords:** PHP, food, safety, molluscan, shellfish, *Vibrio*, virus, irradiation, post-harvest, pasteurization

## Abstract

Post-harvest Processing (PHP) methods are viable food processing methods employed to reduce human pathogens in molluscan shellfish that would normally be consumed raw, such as raw oysters on the half-shell. Efficacy of human pathogen reduction associated with PHP varies with respect to time, temperature, salinity, pressure, and process exposure. Regulatory requirements and PHP molluscan shellfish quality implications are major considerations for PHP usage. Food safety impacts associated with PHP of molluscan shellfish vary in their efficacy and may have synergistic outcomes when combined. Further research for many PHP methods are necessary and emerging PHP methods that result in minimal quality loss and effective human pathogen reduction should be explored.

## 1. Introduction

Oysters, clams, mussels, and scallops are commonly referred to as molluscan shellfish [1]. Molluscan shellfish consume organic material through the flow of water from their aquatic environment by specialized filtration systems. For example, oysters draw in water over their gills using animated cilia, extracting particulate matter in to their mouths [2,3]. Since molluscan shellfish cannot discern what they are consuming there is a possibility that foodborne illness causing bacteria or viruses may be present, or even concentrated, in their tissues [4]. Consequently, eating raw molluscan shellfish involves a higher risk of foodborne illness than those that undergo food processing techniques.

In 1984, the U.S. Food and Drug Administration (FDA) and the Interstate Shellfish Sanitation Conference (ISSC) developed a formal cooperative structure as the National Shellfish Sanitation Program (NSSP) to “promote and improve the sanitation of shellfish (oysters, clams, mussels, and scallops) moving in interstate commerce” [5]. For decades prior, the NSSP has provided guidance associated with the safety of molluscan shellfish. Over the past eight years, the NSSP issued a “Guide for the Control of Molluscan Shellfish” in odd numbered years as a model for molluscan shellfish-producing states [6]. NSSP’s model outlines regulations associated with the classification, harvesting, processing, labeling, storage, handling, packing, shipment, dealer licensure, and aquaculture, in addition to processing methods and labeling requirements for molluscan shellfish harvested during warm water months, contaminated water, or clinical cases associated with *Vibrio vulnificus*, *Vibrio parahaemolyticus*, or other human pathogens found in waters near growing areas [5].

One of the ways suggested to control human pathogens in raw molluscan shellfish by the NSSP are post-harvest processing (PHP) methods, depuration, or wet storage [5]. Examples of PHP methods are thermal processing, freezing, irradiation, high-hydrostatic pressure (HHP), and high-salinity relaying [3,7,8]. If PHPs are used to reduce human pathogens in molluscan shellfish, the dealer must process under a seafood hazard analysis critical control point (HACCP) plan and validate that the process achieves a minimum 3.52 log reduction of viable bacteria and reduces the level of *V. vulnificus* and *V. parahaemolyticus* to non-detectable levels (<30 MPN/g) [5].

Every food processing technique, including the aforementioned PHP methods, causes some change in food quality, ranging from undetectable to inedible. Thermal processing or cooking, for example, is a processing technique applied to raw foods resulting in variable physical, chemical, and biological changes that many consumers consider preferable in comparison, in terms of flavor and texture. With the possible exception of fruit and vegetable consumption, seafood may be the most widely consumed food category in various states of raw to fully cooked [9].

While thermal processing is widely known to be a common and effective method of reducing the risk of consuming pathogenic organisms in food, even gentle heat treatment of many seafood species cause noticeable physical and chemical changes resulting in the reduction of quality [10]. On the other hand, removal of heat (refrigeration or freezing) from seafood results in increased shelf-life and some microbiological reduction [11,12]. However, detrimental quality changes may also occur during frozen storage depending on the rate of freezing, how long seafood is stored, how seafood is packaged, thawing conditions, and intended state of consumption (raw or cooked) [13,14].

## 2. Considerations Associated with Post-Harvest Processing (PHP) of Molluscan Shellfish

Thermal processing and irradiation are effective PHP methods for all foodstuffs given appropriate time, temperature, or exposure for foodborne pathogen reduction. Nonetheless, other food processing techniques may result in higher molluscan shellfish quality, by comparison [2,4,15,16,17,18,19]. For instance, shucked oysters subjected to thermal processing conditions as mild as 50 °C for 5 minutes will yield a 6-log reduction in *V. vulnificus* [16]. Alternatively, freezing oysters at −40 °C and storage for 8–10 weeks achieves a 4- to 5-log reduction in the *V. vulnificus* population [16]. Molluscan shellfish freezing efficacy for pathogen reduction is subject to a number of factors associated with the pathogen’s prolonged exposure to their environment and subsequent adaptability to cold temperatures [14,16,20,21]. Additionally, HHP methods have been very effective in reducing human pathogens in oysters, but vegetative bacteria survive HHP differently and often kills molluscan shellfish [2,3,4,12,22,23]. However, HHP facilitates “commercial shucking” by detaching the adductor muscle from its shell in addition to reduction of *Vibrio* bacteria [2,4,23]. Depuration and high-salinity relaying methods as PHPs tend to be less effective or harder to control [3,24,25,26,27]. 

In the United States, oysters are consumed raw more than mussels, clams, and scallops. Therefore, pathogenic microorganisms in oysters have been studied with more emphasis than other molluscan shellfish. Table 1 outlines data from CDC’s National Enteric Disease Surveillance system called COVIS (Cholera and Other Vibrio Illness Surveillance). In 2013, 290 patients were diagnosed wtihprobable foodborne vibriosis, 149 (51.4%) ate raw oysters, and 21 ate raw clams (7.2%) [28]. Although *V. parahaemolyticus* is perennially the cause of 3–5 times more vibriosis cases than *V. vulnificus*, the number of hospitalizations and deaths associated with *V. parahaemolyticus* is much lower [28,29,30,31,32].

**Figure 1:**
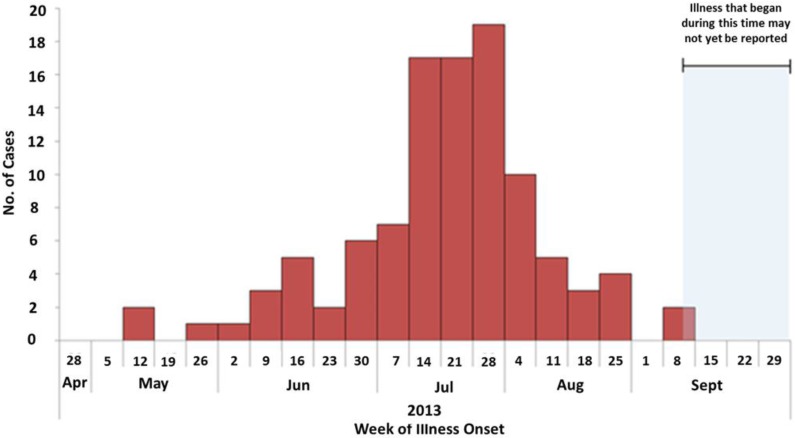
Number of *Vibrio parahaemolyticus* illnesses by week of illness onset [33].

In 2006, the Joint FAO/WHO Expert Consultation working group suggested that effective PHP processing methods should strive to achieve at least a 4-log reduction in *V. vulnificus* levels which would essentially eliminate illnesses if it was universally applied during restricted harvest periods [22]. In seasons where seawater temperatures are highest (May to September), surveillance data in Figure 1 shows higher concentrations of pathogenic bacteria in harvest areas and a greater number of illnesses associated with molluscan shellfish [33].

## 3. Thermal Processing

Thermal processing techniques are thought to have been applied to food since the discovery of fire.Numerous archeological studies provide ample evidence that early man used fire for heat and cooking. Pottery useful for water, fruit, vegetable, grain, and meat heating in an open fire and/or storage are widespread throughout Africa and Central Asian populations at their earliest carbon dating [34]. By thermally processing (heating, cooking, steaming, grilling, *etc.*) many organic materials are made palatable by humankind and reduce many toxigenic compounds [35].

The most common thermal process used to reduce microbiological occurrence and growth in food in a commercial food processing setting is pasteurization. Pasteurization is most commonly used to increase the refrigerated shelf-life of foods and to eliminate certain pathogenic bacteria. For example, to eliminate pathogenic bacteria, liquids have been pasteurized by exposing them to 71.1 °C for 15 s since the early 1900s [36,37]. Other times, thermal processing treatments can be used to extend packaged food quality by eliminating spoilage bacteria. 

As for molluscan shellfish, the major pathogen targeted is *V. vulnificus*. *V. vulnificus* is known to grow between 8–43 °C and *V. parahaemolyticus* grows within the 5–45.3 °C range [38]. FDA recommends boiling live oysters for 3–5 min after the shell opens or steaming for 4–9 min to reduce *V. vulnificus* [39]. FDA also suggests discarding any oysters, mussels, or clams that do not open during cooking [40]. Other methods for cooking molluscan shellfish include frying and baking. Federal agencies suggest frying shucked oysters at 191 °C and baking oysters at 232 °C for 10 min [41].

## 4. High Pressure Processing

High hydrostatic pressure (HHP) is a food processing method studied for more than three decades, although the concept of high pressure preserving food by microorganism reduction was first postulated in 1899 [42,43,44,45,46,47,48,49,50]. HHP is also described as ultra-high pressure (UHP) processing. HHP methods applied to seafood were first introduced in 1999 [43]. HHP theorizes a condition of equal, but high (200–700 MPa), pressure in a vessel containing food for human consumption [4]. Under HHP, all metabolic tissues are processed in a closed vessel that applies high pressure equally and at the same time to minimize moisture loss and eventual product quality, while destroying biological cells. *Vibrio* spp. appear to be more susceptible to HHP than other bacteria found in oysters, with greater than a 6-log reduction pressurized at 250 MPa (25 °C) for 15 min or 300 MPa (25 °C) for 5 min [44].

There are several other iterations to high pressure food processing that include supercritical carbon dioxide processing (scCO_2_), where carbon dioxide changes phase from gas to liquid at high pressures. scCO2 processing of oysters showed similar results in terms of quality and reduction of bacteria and *V. vulnificus* inactivation when compared to HHP [43]. 

The preparation of raw molluscan shellfish is labor intensive in that they are either partially shucked or completely separated by hand. HHP processes at pressures of 250 to 300 MPa (25 °C) also provides potentially favorable separation of the adductor muscle from their shells in preparation for human consumption [44,45,46,47]. HHP is currently the favored method of post-harvest processing of molluscan shellfish in the Southeastern U.S. with several facilities currently in operation. Up to 700 pounds of shucked oysters can be HHP treated per cycle in some facilities serving the oyster production from the Gulf of Mexico [48]. 

HHP is also known to inactivate human viruses found in the tissues of shucked oysters, mussels, and clams [10,23,42,45,49,50]. Viruses commonly implicated in human foodborne illness from molluscan shellfish are norovirus and hepatitis A [49]. In addition to high pressure level and other considerations, such as pH, temperature, or food source, pressure-related virus inactivation has variable outcomes [42,49,50]. If most foodborne viruses opportunistically find their way to a human host, only a scintilla of viral particles may cause foodborne illness symptoms where they may be replicated and shed either in or out of the food supply with more persons affected. In the case of molluscan shellfish, viruses may accumulate in filter feeders (as much as 1000-fold from contaminated waters) and commonly survive through harvest, transport, and presentation for consumption [49,50,51]. Virus inactivation in all foods can be complicated because they are environmentally stable with the ability to survive through many food safety related hurdles, including resistance to low pH, surfactants, and sanitizers [49]. Furthermore, some foodborne viruses are more resistant to food processing than some pathogenic bacteria, including HHP.

Studies published by Lou, *et al.* in 2015 provide a thorough review on virus inactivation efficacy in various food matrices [50]. Hepatitis A virus in oysters undergoing HHP showed a 6-log reduction at 350–400 MPa held under refrigerated temperatures near 10 °C for in only one minute [42]. Live oysters inoculated with human norovirus held under 400 MPa for 5 min are reduced by 1.3–4.0 logs between 5–6 °C [49,50]. Raw oysters undergoing HHP treatments at pressures as high as 400 MPa are considered to be of good quality, although commercial molluscan shellfish HHP treatments fall within the 275–300 MPa range and may not reduce the viral load to an adequate level in contaminated waters [49,50,51].

Product banding (*i.e.*, red rubber bands heat-shrunk to cover the shell before and after production) is required to substantiate that shellstock is processed by HHP to consumers and/or purveyors [48]. Without product banding, it is likely that the molluscan shellfish will open during HHP processing. Observable open shells are typically a sign of molluscan shellfish death in raw unprocessed product. Packaging bands are only heated from the outside of shells to necessitate their function of closure during the process, and are not considered in the reduction of pathogens for molluscan shellfish.

## 5. Heat-Cool Pasteurization

In the mid-1990s, NSSP called for methods to devise a post-harvest process that could be applied to raw Gulf of Mexico oysters to reduce *V. vulnificus* [48]. In 1995, AmeriPure Processing Company, Inc. (Franklin, LA, USA) patented a PHP using minimal heat application in shelled oysters to destroy *V. vulnificus* immediately followed by cooling. This process requires a washing step with potable water, followed by immersion in a 7500-gallon tank containing water maintained at 53 °C tank for 24 min, then transfer to a 5500-gallon (4 °C) tank for 15 min, thereby processing up to 10,500 shelled oysters per cycle. AmeriPure named the patented process Heat-Cool Pasteurization (HCP) [52]. 

## 6. Irradiation

Food irradiation has been studied for decades [19]. In what may be considered the most prescient of irradiation applications was the use in NASA’s space program. The U.S. FDA approves the use of irradiation as a PHP for oysters and has been validated by a number of researchers [19,53,54,55,56,57,58,59,60,61]. 

The main challenge involved with irradiating molluscan shellfish is regulatory and associated with interstate commerce regulations [61]. For example, if packages are pre-labeled as irradiated and travel to another state for irradiation processing, the product would be in violation of federal labeling laws as it crosses state lines prior to irradiation. The FDA has considered enforcement discretion of this law under certain conditions, such as written and signed agreements between the irradiation processing establishment and the primary processor. For FDA enforcement discretion to apply, both parties should have an approved HACCP plan allowing for sealed trucks, palletizing specifications, labeling of molluscan shellfish pallets to be irradiated, and proper record keeping [48]. Product banding would also need to be considered, as irradiation will cause shell opening at certain exposure levels (5.5 kGy) and for labeling purposes [59]. 

Oyster irradiation is quite efficient, in that an entire tractor trailer can be treated in 60 min [48]. Due to the speed associated with irradiation PHP, oysters do not need to be stored by the irradiation facility. However, the limited amount of irradiation facilities and their proximity to molluscan shellfish harvest sites presents challenges for wide-spread use by molluscan shellfish harvesters. 

## 7. Quick Freezing/Frosting

PHPs that rapidly remove heat from molluscan shellfish are said to have been Individually Quick Frozen (IQF), Quick Frozen, Cryogenically Frozen (CF), or Frosted. First investigated in the 1980s, freezing food for one month results in up to 1- to 2-log reductions in bacteria [62,63,64,65,66,67]. Six months of frozen storage at −10 °C resulted in 4.55-log reduction of *Vibrio parahaemolyticus* in inoculated raw oysters [63]. Freezing processes to reduce bacteria in oysters are typically sent through a freezing tunnel, oysters are subjected to temperatures of −10 to −80 °C, before being sprayed with water that promotes a glaze of ice on the interior of the half-shell and meat [48,61]. Although some microbiological reduction is apparent during initial freezing, it is necessary to hold half-shell oysters treated in this manner for 12 weeks to achieve necessary levels of *V. vulnificus* (<3 MPN/g) [16]. The wide range of necessary time in frozen storage is a function of initial *V. vulnificus* load, where oysters harvested from warm water typically have higher initial levels than oysters harvested from cooler water temperatures. ISSC suggests that oysters harvested from warm water must be stored under freezing conditions for an elongated period of time that negatively affects quality by loss of oyster meat flavor, texture, and appearance [48,61]. ISSC also notes, however, that molluscan shellfish processed by HHP synergistically allows them to withstand long-term freezing processes better than raw oysters based on unpublished industry observations [22]. 

## 8. High-Salinity Treatment/Relaying

Investigations associated with *V. vulnificus* and *V. parahaemolyticus* survival in higher salinity waters have been ongoing since the 1990s [26,27,68,69,70,71,72,73,74]. Using a technique called relaying, oysters can be transferred to other higher salinity waters (30–35 ppt) than those of harvest waters (8–15 ppt) to achieve a reduction in pathogenic bacteria (10 MPN/g *V. vulnificus*) in as little as 28 days [26,27]. High-salinity relaying is also used in molluscan shellfish transfer to more controlled environments, such as land-based tanks with similar results [71,72,73]. High-salinity relaying is a promising PHP, but may be difficult to control in natural relaying environments [74]. 

Although high-salinity relaying procedures seem effective in controlled systems, *V. vulnificus* reduction validation still remains questionable for entire populations of oysters harvested in certain geographical areas, and additional research is required to determine efficacy of the process [68,69,70,71,72]. 

## 9. Depuration

The National Shellfish Sanitation Program (NSSP) defined depuration as “the process of using controlled aquatic environment to reduce the level of bacteria and viruses that may be present in [live] shellfish harvested from moderately polluted [restricted] waters to such levels that the shellfish will be acceptable for human consumption without further processing” [75]. There is ample historical evidence that molluscan shellfish depuration has been a concept for well over 100 years [76]. In fact, the United States Public Health Service (USPHS) demonstrated oyster depuration methodology in 1916 [76]. 

Coupling depuration methods with sanitizing controls, such as chlorine, iodophors, ultraviolet (UV) light, or ozone, can be effective in disinfecting seawater [77,78,79,80,81]. However, with the exception of UV light treatment, disinfection compounds become toxic to live molluscan shellfish [78]. 

Another depuration application is to reduce or eliminate off-flavors formed by *Actinomyces* and blue-green algae [82]. Depuration recirculating systems are also commonly used in land-based aquaculture, although metabolic end product concentration, biological oxygen demand, shifting pH, and waste accumulation are common limitations of use. Depuration is largely ineffectual in reducing *V. vulnificus* in oysters [3,79]*.* However, depuration with microbiological monitoring controls is also quite effective in the reduction of pathogenic bacteria in bivalve mollusks [77,78,79,80,81].

## 10. PHP Reduction of Other Potential Food Safety Issues Associated with Raw Molluscan Shellfish Consumption 

Human consumption of raw molluscan shellfish results in a number of food safety related issues. *Vibrio vulnificus, Vibrio parahaemolyticus, Salmonella, E. coli*, Norovirus, and potential exposure to toxic chemicals have been the major concerns over the last 20 years [83,84,85,86,87,88,89,90,91,92]. Certain human pathogens of interest to molluscan shellfish processors, like *V. vulnificus* and *V. parahaemolyticus* species that may cause human health issues are typically considered to be source of food safety issues. Other causes of foodborne illness symptoms (nausea, vomiting, *etc.*) from fecal-oral route viruses in as little as 12 h after consumption of raw contaminated molluscan shellfish is also a food safety consideration. Viral organisms are inactivated by minimally destructive PHP methods that only mildly influence quality [48,50,51]. Molluscan shellfish harvest areas are subject to waste dumping controls from state and local agencies but any human waste seawater contamination event impacts the safe consumption of raw molluscan shellfish [5].

Another consideration to food safety and quality is that oysters, for example, have difficulty surviving PHP methods. Traditionally, restaurants serving raw or cooked oysters rely on simple observations, like open or closed shells before shucking, to determine quality and to some extent, safety. To limit confusion, independent education outreach programs associated with product banding, labeling, interstate commerce, potential changes in quality, and enhancements in safety as a result of PHPs should be provided to businesses serving raw molluscan shellfish. 

## 11. Conclusions

There are many aspects of post-harvest processing that provide an increase in safety to consumers of molluscan shellfish. However, consideration of expense, availability of processing equipment or toll processors, and quality degradation should be considered. Although all of these methods show reduction of potential pathogen contamination, quality and edibility of the processed products continue to be major concerns for the molluscan shellfish industry.

Until the understanding and necessity of scientifically-based research for safe and high-quality seafood products has been conveyed to the general public and necessary buy-in from raw molluscan shellfish consumers, PHP molluscan shellfish products will likely have concerns attached to them and may not be worth the cost to “minimally process” oysters, clams, mussels, and scallops.

Many consumers consider most raw seafood to be healthful and safe for consumption. The scientific community must continue to study seafood that consumers prefer to consume raw. Outreach efforts may also subjugate misconceptions about PHPs and portray the very real potential of human illness or death if molluscan shellfish are not processed when harvested from warm or contaminated waters. With noticeable increases in seawater temperatures, there is the potential for greater concern with raw molluscan shellfish consumption. *Vibrio vulnificus* and *Vibrio parahaemolyticus* could be found in higher numbers for longer periods of time, not to mention emerging issues that we may not have contemplated. Thankfully, we have the food processing technology to provide products that inactivate pathogenic organisms while maintaining similar quality compared to raw molluscan shellfish. The PHP of molluscan shellfish should be seriously considered by molluscan shellfish processors now and in the future. Additionally, the scientific community should consider combining more PHP technologies for potential symbiotic pathogen reduction measures yielding increased quality of molluscan shellfish products. 

## Figures and Tables

**Table 1:** Number of vibriosis patients with *V. parahaemolyticus* and *V. vulnificus* in the United States (2009–2013) [28,29,30,31,32].

# of patients (*% of total*)	2009	2010	2011	2012	2013
*V. parahaemolyticus*	386 (*46.8*)	421 (*45.4*)	334 (*39.2*)	431 (*45.7*)	594 (*50.5*)
*V. vulnificus*	107 (*13.0*)	133 (*14.3*)	113 (*13.2*)	119 (*12.6*)	137 (*11.6*)
*Vibrionacea* Total	825 (*59.8*)	927 (*59.8*)	853 (*52.4*)	944 (*58.3*)	1176 (*62.2*)

**Table 2:** Number of vibriosis patients who reported eating seafood, consumed raw oysters, or raw clams in the United States (2009–2013) [28,29,30,31,32].

# of patients (*% of total*)	2009	2010	2011	2012	2013
Vibriosis Patients	825	927	853	944	1176
Reported Eating a Seafood Item	236 (*28.6*)	182 (*19.6*)	184 (*21.6*)	211 (*22.4*)	290 (*24.7*)
Consumed Raw Oysters	106 (*12.8*)	72 (*7.8*)	106 (*12.4*)	104 (*11.0*)	149 (*12.7*)
Consumed Raw Clams	16 (*1.9*)	11 (*1.2*)	9 (*1.1*)	21 (*2.2*)	21 (*1.8*)

## Abbreviations

The following abbreviations are used in this manuscript:

CC-BY: Creative Commons by Attribution
CDC: United States Centers for Disease Control and Prevention
CF: Cryogenically Frozen
CFU: Colony Forming Units
COVIS: Cholera and Other Vibrio Illness Surveillance
DOAJ: Directory of open access journals
DOI: Digital Object Identifier
Epi Data: Epidemiological Data from the Centers for Disease Control and Prevention
FDA: United States Food and Drug Administration
HACCP: Hazard Analysis Critical Control Point
HCP: Heat-Cool Pasteurization
HHP: High Hydrostatic Pressure
IQF: Individually Quick Frozen
ISSC: Interstate Shellfish Sanitation Conference
MDPI: Multidisciplinary Digital Publishing Institute
MPa: Mega Pascal
MPN: Most Probable Number
NASA: United States National Aeronautical and Space Administration
NSSP: National Shellfish Sanitation Program
PHP: Post-Harvest Processing
ppt: parts per thousand
U.S.: United States of America
USPTO: United States Patent and Trade Office
*V.*: *Vibrio*
